# Subpial Hemorrhage in an Adult Male

**DOI:** 10.7759/cureus.28404

**Published:** 2022-08-25

**Authors:** Shyam H Bhatt, Thomas V Kodankandath

**Affiliations:** 1 Neurology, Virginia Tech Carilion School of Medicine, Roanoke, USA; 2 Neurology, Carilion Clinic, Roanoke, USA

**Keywords:** neuro mri, neuro radiology, neuro-vascular, intracerebral hemorrhage, adult neurology

## Abstract

Subpial hemorrhage (SPH) is a rare entity that may often be overlooked in favor of more common intercranial bleeds, especially in adults. Our case demonstrates classical imaging findings seen in SPH in an adult male. SPH is poorly understood, with no identified clinical syndrome or symptomology, and further identification and classification of SPH are necessary to develop a more thorough understanding of its pathophysiology and potential treatment protocols.

## Introduction

Intracranial bleeding is a well-studied phenomenon that can be divided up into central and peripheral causes for treatment purposes [1]. Central bleeding includes intraventricular and parenchymal hemorrhage. Peripheral bleeding is generally defined by its location around layers of the meninges, consisting of the pia mater, arachnoid mater, and dura mater; which surround the cerebral cortex and spinal cord. Most commonly, peripheral bleeding occurs in the epidural, subdural, or subarachnoid spaces, however subpial hemorrhage (SPH) has also been reported.

Despite causing neurological injury that may persist into perpetuity, we have little clinical understanding of SPH. Patients often present with seizure-like activity and radiographic findings that may mimic other cerebral hemorrhages [2]. Currently, all data available is in the form of case reports or case series, thus there is great variation in patient diagnosis, treatment, and prognosis. Here we describe SPH in a 58-year-old man, in an effort to better describe SPH and further this growing body of knowledge.

## Case presentation

We present a 58-year-old man with a history of type 2 diabetes mellitus, dyslipidemia, and tobacco abuse presenting as a transfer from a community hospital where he was diagnosed with intraparenchymal hemorrhage. He had been complaining of a tension-like headache for several days, and his wife had found him outside confused, having forgotten where he was going in the middle of their yard. Before the presentation, his wife reported seizure-like activity, in which the patient was unresponsive, with violent jerking of his extremities, characterized as a tonic-clonic seizure. She reported him to be drowsy and confused afterward and had a left-sided weakness when EMS arrived. Upon presentation to the community hospital emergency department, the patient had another episode of tonic-clonic activity, after which he was intubated and given levetiracetam. Head CT was read as a moderate subarachnoid hemorrhage (SAH) along the right temporoparietal region with vasogenic edema with sulcal effacement. Patient vitals upon arrival was significant for a blood pressure of 88/55 and O_2_ saturation of 98% on a ventilator. Upon admission, lab abnormalities (Table 1) included an elevated BUN of 27 mg/dL, elevated creatinine of 1.51 mg/dL, and elevated blood glucose from baseline at 189 mg/dL. GFR was low, but at baseline level for the patient, BMP lab values from admission are included in Table 1.

**Table 1:** BMP lab values of the patient upon admission Elevated BUN, Cr, and blood glucose levels are not surprising given the patient’s history of poorly controlled type 2 diabetes and poor renal function. While elevated, these values were deemed unlikely to be the causes of the patient’s presentation with seizures.

Lab	Value	Reference range
Sodium	137	135-145 Mmol/L
Potassium	4.8	3.8-5.3 Mmol/L
Chloride	101	95-107 Mmol/L
CO_2_	22	21-31 Mmol/L
Urea nitrogen	27	6-20 Mmol/L
Creatinine	1.51	0.5-1.4 mg/dL
Blood glucose	189	70-99 mg/dL
Calcium	9.0	8.5-10.7 mg/dL
Anion Ga	14	8-18 Mmol/L
Osmolality Calc.	294	278-305 Mos/kg
Bun/creatinine	17.9	7-20
GFR	49	>60 mL/min/1.73m^2^

**Figure 1:**
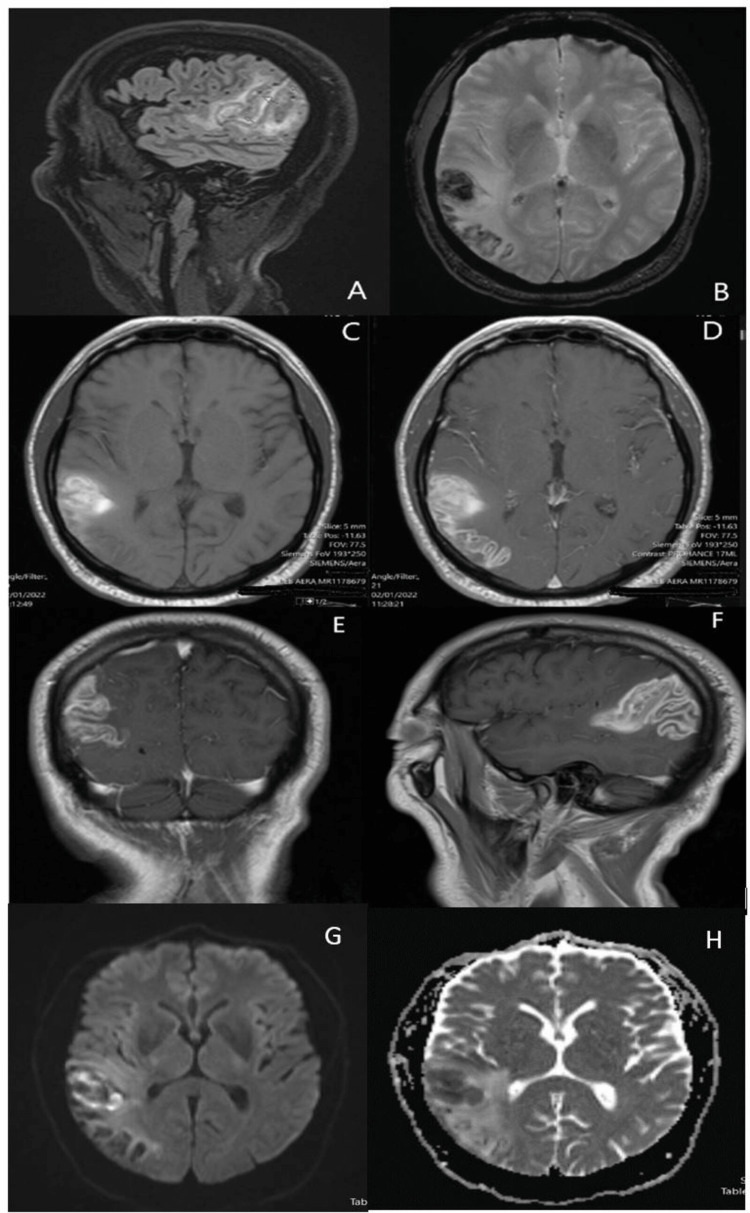
MRI findings Fine, planar hyperintensity (arrows) seen on 3D Flair from dorsal cephalad to ventral supermarginal gyrus (A), T2-weighted hemosiderin view (B) of hemorrhage, axial T1-view of insult on dorsal one-third of temporal gyrus, dorsal posterior ramus of supramarginal gyrus, and angular gyrus pre- (C) and post- (D) contrast, showing hypointensity in areas with vasogenic edema. T1 post-contrast coronal view (E) and sagittal view (F) showing vasogenic edema surrounding hemorrhage in superolateral occipital lobe, with hemorrhage extending along gyrus without intracerebral extension. DWI images showing hyperintensity with surrounding edema (G) and ADC (H) showing R-sided edema extending intracortically.

**Figure 2:**
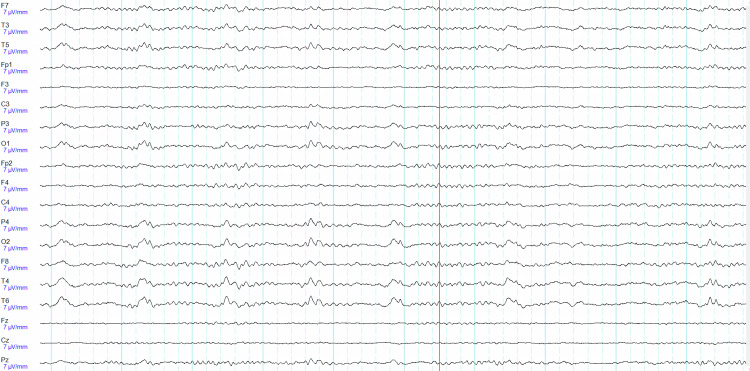
EEG acquired at time of presentation due to multiple seizures reported by patient’s wife and R posterior temporal dominant periodic lateralized discharges, and otherwise normal activity.

One day after admission, the patient remained intubated and so their mental status was unable to be assessed. Neurological examination including assessment of cranial nerves, reflexes, sensation, and motor strength showed no abnormalities. Repeat head CT was read as a stable SAH with adjacent edema in the right parietal lobe. However, an MRI on the same day (Figures 1A-1H) was able to better elucidate the nature of this bleed. Hemorrhagic insult was noted on the dorsal one-third of the superior temporal gyrus, on the dorsal aspect of the posterior ramus of the supramarginal gyrus, with some apical sparing; the angular gyrus, and the superolateral aspect of the occipital lobe. Intrinsic T1 hypointensity denoting vasogenic edema with non-mass-like enhancement was seen in these processes. A fine, planar hyperintensity was also seen on 3D Flair from the dorsal cephalad to the ventral aspects of the above-mentioned gyrus. While originally thought to be a hemorrhagic conversion of an ischemic stroke, it differed in the hyperintensity seen on Flair and cortical edema and would thus be an irregular presentation of a relatively common ailment. Instead, these features suggest a classical picture of the seldom-seen SPH. EEG (Figure 2) showed R posterior temporal dominant periodic lateralized discharges.

## Discussion

First reported in 1972, SPH describes an intracranial bleed that resides in between the pia mater and cortical tissue. Of the reported cases, most have been in neonates [3]. Imaging findings may be difficult to interpret without using flair imaging and having experts in brain imaging reading the film. On CT scans, SPH may be hard to differentiate from other etiologies due to a lack of dynamic fluid imaging. MRI scans may reveal bleeds that do not conform to a singular arterial territory or along the cortical surface. Lack of centripetal radiation of surrounding edema may also provide a clue to a potential SPH. Without adequate exposure to and training in neurological imaging, these subtle findings can be difficult to identify as SPH. In differentiating SPH from other types of bleeds, this cortically adjacent injury may be used as a preliminary determining factor in identifying SPH [4].

Most reports of SPH have been in the frontal lobe, less frequently occurring in temporal and parietal regions, with some instances of cerebellar hemorrhage [5]. Due to the impermeability of the pia mater, blood was not found in the CSF in any case report. This may allow for a lumbar puncture to be a potential diagnostic tool for separating SPH from other cranial hemorrhages that may present with xanthochromia.

The etiology of SPH is not known, however, several mechanisms have been proposed. Cerebral vein thrombosis may lead to elevated venous pressures causing vascular rupture, but few presentations of SPH have been seen with thrombotic activity. Additionally, Sylvian fissure SPH is often seen in aneurysmal SAH with clot formation in the inferior limiting sulcus and an increased degree of clotting has been shown to be associated with the presence of Sylvian SPH [6]. However, another study found subsequent development of a hematoma within the Sylvian space following SPH, which may indicate the relationship working in reverse [7]. Thrombosis was considered on the differential on our patient, as opposed to a potential hemorrhagic conversion of ischemic stroke [8], but lack of laboratory support and clinical findings kept this lower on the differential. Cerebral vasospasms in the subpial region may also play a role in SPH. Risk factors of SPH are similar to that of other cerebral hemorrhages, and management of hypertension, hyperlipidemia, and tobacco abuse will likely prove imperative in preventing SPH. Previous literature on neonates has demonstrated that risk factors for that age group include cardiorespiratory failure, hypoxic-ischemic encephalopathy, and coagulopathy [9]. Fragile cerebral vasculature of infants is another potential risk factor, as mechanical forces have been shown to cause SPH in neonates, while this has never been reported in the adult population [10].

Recognition of bleed location on imaging plays an important role in diagnosis and prognostic determination. Classical imaging results consist of blood pooling along the cortical surface [11], rather than spreading along the convexity as seen in SAHs. Underlying cortical edema or restricted diffusion may also be seen, as well as hyperintensity of the blood [12], due to lack of mixing with CSF. These features may help distinguish SPH from SAH, helping identify these rare entities and invite further study [13]. While no curative treatment has been identified, supportive treatment for patients in distress is indicated, as well as seizure prophylaxis for those presenting with epileptiform activity. Surgical correction has not been used to treat SPH, as the injured vasculature in SPH is small vessels that hemorrhage non-intervention volumes of blood.

## Conclusions

The most common presentation for patients with SPH has been seizures, with headaches, ataxia, and aphasia also seen. SPH is a rare entity that is poorly understood in contemporary neurological medicine, and as such, no unifying syndrome for SPH symptoms has been designated. Overall, subacute peripheral intracranial bleeds with unclear bleed etiologies must be investigated more thoroughly with testing including lumbar punctures to rule out SPH.

This case report describes a temporo-parietal SPH in an adult man, the first known such report. This is one presentation of SPH and the medical decision-making, conclusions, and treatment involved in caring for this patient. Adult SPH may often be confused with similar-looking intracranial bleeds, and identification of SPH is vital to isolating any potential syndromes that may result from SPH and their treatment.
